# Rational medication management mode and its implementation effect for the elderly with multimorbidity: A prospective cohort study in China

**DOI:** 10.3389/fpubh.2022.992959

**Published:** 2022-09-06

**Authors:** Qi Tang, Litao Wan, Jing Lu, Wenhui Wu, Huanyun Wu, Zhenwei Liu, Sitang Zhao, Chengyue Li, Gang Chen, Jun Lu

**Affiliations:** ^1^School of Public Health, Fudan University, Shanghai, China; ^2^China Research Center on Disability, Fudan University, Shanghai, China; ^3^Key Laboratory of Health Technology Assessment, National Health Commission, Fudan University, Shanghai, China; ^4^Shanghai Jinshan District Health Service Management Center, Shanghai Jinshan District Municipal Health Commission, Shanghai, China

**Keywords:** elderly, chronic disease, medication management, health education, family physicians, public health challenge

## Abstract

**Background:**

As one of the countries with the most serious degree of aging, the incidence of potentially inappropriate drug use among the elderly is as high as 30. 4% in Chinese communities, and the lack of effective medication management and poor medication compliance at home are the main factors. Given these situations, we constructed a Rational Medication Management Mode based on family physician service, carried out an empirical research and evaluated the implementation effect.

**Methods:**

A prospective cohort study was conducted from September to December 2021 to analyze the implementation effect of the Rational Medication Management Mode by comparing the outcome indicators between the intervention group and control group. The primary outcome of this study was medication number and polypharmacy (taking 5 or more medications) at 90 days. The secondary outcomes included the situation for behavioral self-management and knowledge-belief-behavior of rational medication use.

**Results:**

A total of 618 elderly patients (309 in the intervention group and 309 in the control group) with multimorbidity were included in this study, those were all available at follow-up at 90 days. At 90 days, the number of medications was achieved by 3.88 (1.48), and patients with polypharmacy were reduced by 59.55% in the intervention group, having a significant difference compared with the control group (*P* < 0.001). Patients with medication reminders, intermittent medication and adverse drug reactions were achieved in 294 (95.15%), 47 (15.21%), and 51 (16.51%) respectively in the intervention group (*P* < 0.001). The knowledge, belief, behavior security and behavior compliance of rational medication use of elderly patients were all greatly improved in the intervention group at 90 days (*P* < 0.0001).

**Conclusion:**

The Rational Medication Management Mode based family physician service, which provides the support of manuals and pillboxes, can decrease the elderly patients' number of drugs with multimorbidity, reduce the incidence of polypharmacy, enhance behavioral self-management, increase the knowledge and belief of rational medication use, and improve the security and compliance of medication usage behavior. In order to provide a practical basis for rational medication management of elderly patients with multimorbidity under the background of long-term prescriptions in China.

## Introduction

The elderly have a high prevalence of chronic diseases, and often suffer from multimorbidity, requiring long-term medication (1). As the elderly often repeat treatment in multiple departments and hospitals, in this process of medical transfer and transition, several specialists may prescribe repetitive medicines using disease-specific guidelines due to the lack of information interconnection, resulting in patients' repeated medication. Relevant studies have found that the elderly visit 4 different medical institutions and 8 different doctors each year on average, and the average dosage is more than 5 times that of young people (2, 3). The combined use of these medicines may cause harm to patients if there is no professional drug integration (4, 5). In addition, irrational use of medications can stimulate inappropriate patient demand, and may lead to a loss of patient confidence in the health system (6).

The World Health Organization (WHO) put forward the concept of rational medication use in 1985, which refers to patients receiving medications appropriate to their clinical needs, in doses that meet their requirements, for an adequate period, and at the lowest cost to them and their community (6). Irrational use of medications in a way that is not compliant with rational use as defined above, which included polypharmacy, inappropriate use of antimicrobials, over-use of injections, inappropriate self-medication, *etc*. Worldwide, more than 50% of all medications are inappropriately prescribed, dispensed, or sold, while 50% of patients fail to take them correctly (6). Technology tools, such as electronic personal health records, mobile applications, paper versions of medication record forms, *etc*, could improve both patient experience and medication adherence, and potentially enable patients to be active participants in medication reviews (7). Some studies have revealed that technology/tool-based medication management modes (8–10), such as the doctor-patient participatory mode and the pharmacist-leading home-dwelling mode, have achieved good results in the management of hypertension, diabetes, osteoporosis and other chronic diseases (11, 12), which helps to standardize the prescription behavior of medical service providers and improve patients' medication compliance and health outcomes. Moreover, studies estimated that a total of USD$18 billion, 0.3% of the global total health expenditure, could be avoided by appropriate medication management (13).

As one of the countries with the most serious degree of aging, there are 182.17 million elderly people aged 65 and over who suffer from at least one chronic disease in China, with an average of 3.1 diseases per capita (14, 15). Relevant study shows that the incidence of potentially inappropriate drug use among the elderly is as high as 30.4% in Chinese communities, and the lack of effective medication management and poor medication compliance at home are the main factors (16). A study demonstrates that <50% of patients with chronic diseases can still strictly take medications at home according to doctor's advice 1 year after discharge (17). Since more than 90% of the elderly always live at home, and the family physician system has been developed in China for more than 10 years, community healthcare centers have become a key link and an important way to strengthen rational medication management at home for the elderly (18). However, previous studies have shown that primary medical institutions in China are incompetence of pharmaceutical personnel and weak in pharmaceutical care, making it difficult to realize whole-process pharmaceutical care for elderly patients with chronic diseases at home. The elderly themselves also have problems, such as lack of drug knowledge, incorrect drug concepts, poor drug compliance, and lack of supervision from doctors in-home medication, which emphasizes the problem of irrational drug use (19).

Given these situations, we constructed a rational medication management mode based on family physician service, carried out empirical research and evaluated the implementation effect, improving elderly patients' health outcomes and providing a reference for the policy-making of rational medication management for the elderly in China.

## Materials and methods

### Rational medication management mode in this current study

Based on family physician service, the Rational Medication Management Mode in this current study specifically refers to the joint participation of family physicians, pharmacists, family physician assistants, nurses, elderly and their family members to carry out medication reconciliation and drug use guidance through medication management manual and pillbox.

The medication management manual (tool) in this study contained “Basic information”, “Diagnosis and treatment”, “Medications list”, “Health care products list”, “Medications record weekly table”, “Health-related index record weekly table”, “Self-evaluation of medication use compliance” and “Medication management evaluation from family physician” eight functional modules, which are jointly recorded by the family physician team, elderly patients and their families (see Supplementary Table 1 for framework and content).

**Figure 1:**
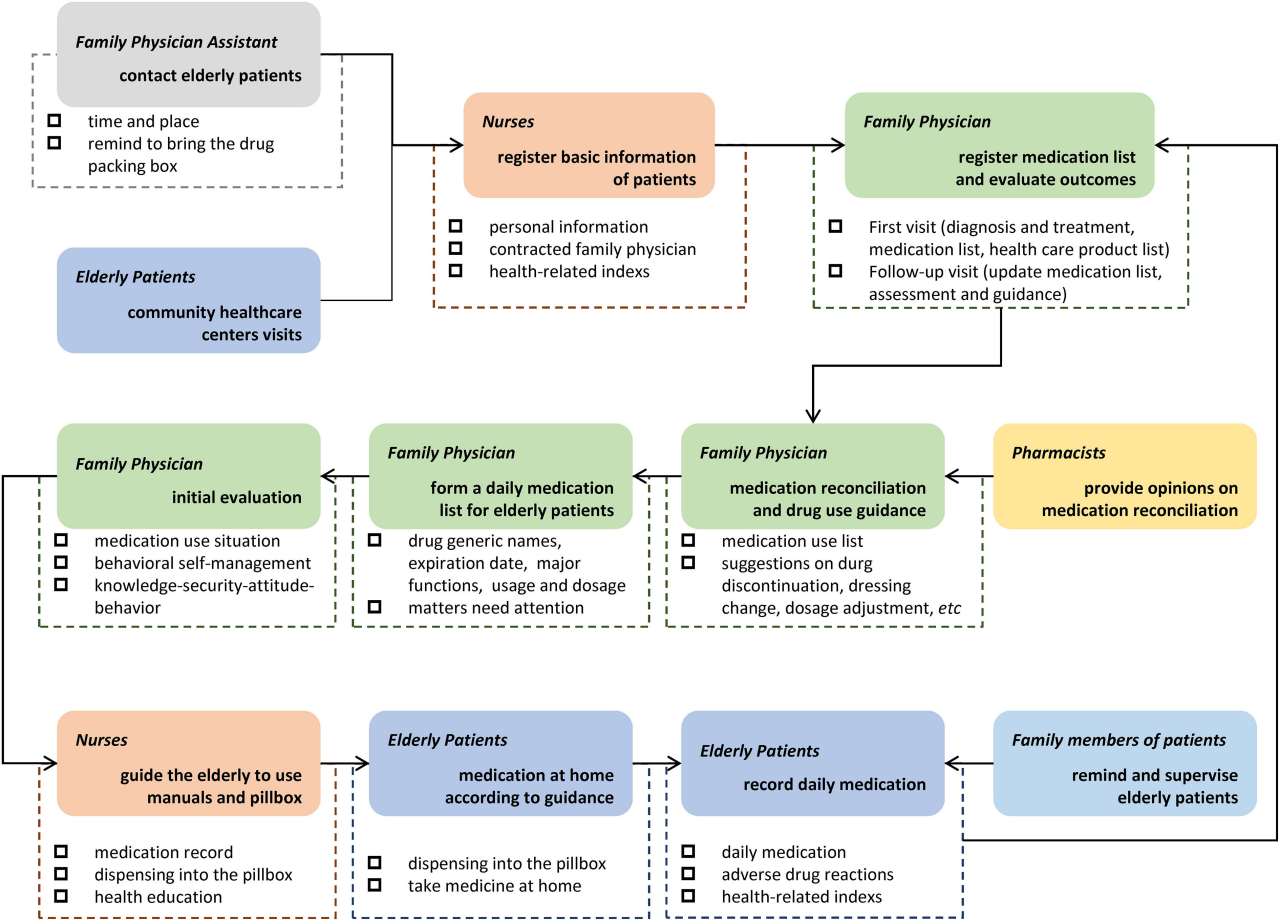
Operation process of Rational Medication Management Mode. Based on family physician service, the mode in this current study specifically refers to the joint participation of family physicians, pharmacists, family physician assistants, nurses, elderly and their family members to carry out medication reconciliation and drug use guidance through medication management manual and pillbox.

The operation process of Rational Medication Management Mode in this current study is shown in Figure 1.

### Empirical study design

In this study, two community healthcare centers were respectively selected in the central-city and countryside areas of Shanghai, divided into an intervention group and a control group. A prospective cohort study was conducted from September to December 2021 to analyze the implementation effect of the Rational Medication Management Mode by comparing the outcome indicators between the two groups.

Shanghai is a typical megacity in China, with developed economy and deep aging population. In 2011, Shanghai began to pilot the family physician system in community healthcare centers. In 2015, the “1+1+1” contract service mode was established, proposing to add pharmacists to the family physician team. In 2016, Shanghai took the lead in piloting the training of community clinical pharmacists. This series of explorations have provided a guarantee for the development of Rational Medication Management Mode for the elderly.

The selected community healthcare centers have the following characteristics: (*i*) complete functions with medication management, health monitoring, health education, *etc*; (*ii*) the family physician signing rate of the elderly aged 65 years old is more than 90%; and (*iii*) the family physician team organized well, equipping with general practitioners, nurses, pharmacists, family physician assistants, *etc*.

**Figure 2:**
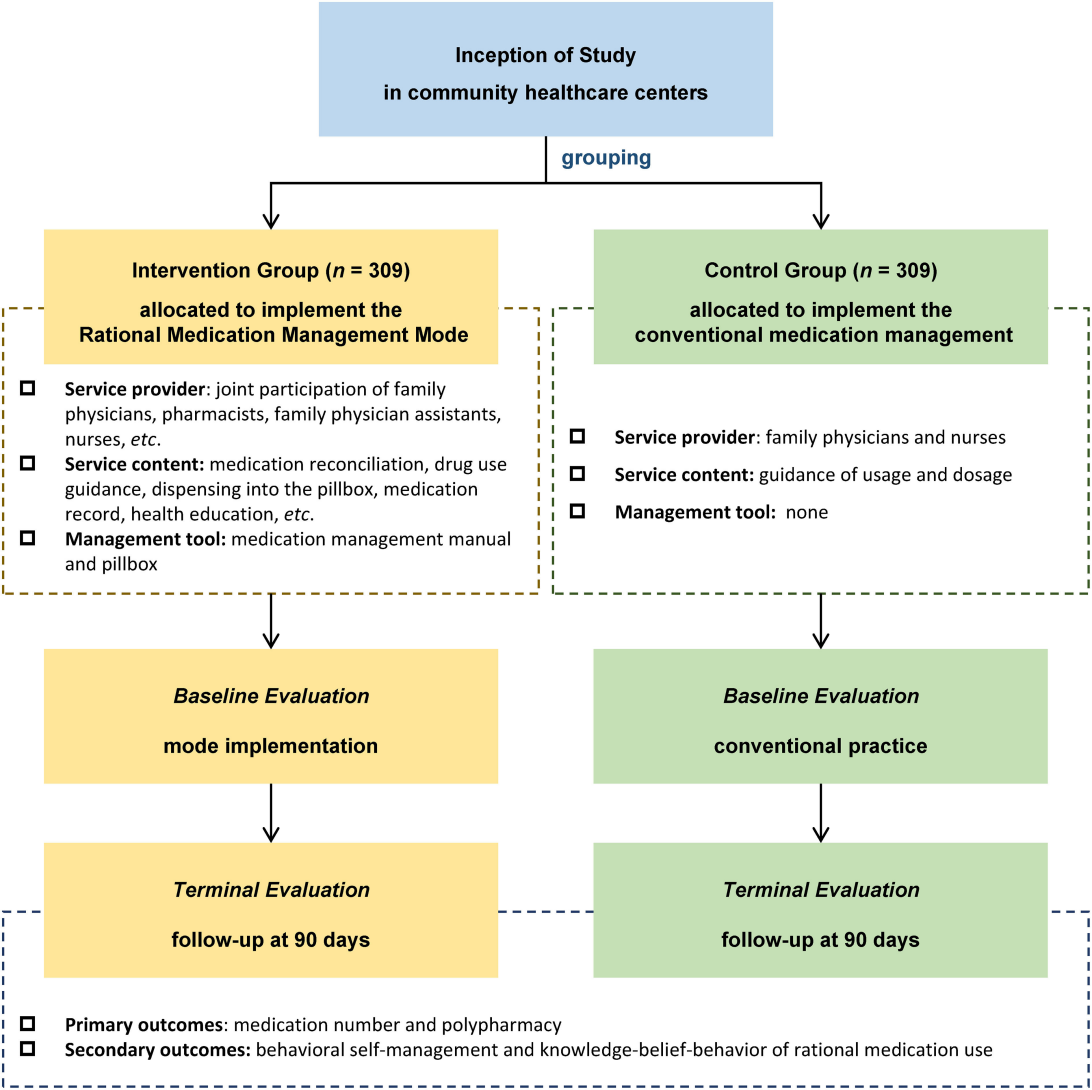
The flow chart of implementation effect research. A prospective cohort study was conducted from September to December 2021 to analyze the implementation effect of the Rational Medication Management Mode by comparing the outcome indicators between the intervention group and control group. The primary outcome of this study was medication number and polypharmacy (taking 5 or more medications) at 90 days. The secondary outcomes included the situation for behavioral self-management and knowledge-belief-behavior of rational medication use.

The target sample size is based on the calculation formula which compares 2 means with two groups:


$$\begin{array}{l} n\;=\frac{{\left(Z_{\alpha \;}+Z_{\beta }\right)}^{2}*2\sigma^{2}}{\delta^{2}} \end{array}$$

where α is 0.05, Z value is two-sided, so Z_α_ = 1.96; power of test is 0.9, so Z_β_ = 1.28. According to the pre-survey, the standard deviation (SD) of an average number of drugs was 1.71 (σ), and the difference value between the two groups was 0.49 (δ), so the calculated sample size was 256 per group. In this study, convenience sampling was used to select contracted elderly patients with comorbidity by family physician, and the propensity score matching was used to perform 1:1 matching on the baseline data of age, gender, living areas, marital status, education, living pattern, monthly income, insurance type, number of diseases and number of medication. Ultimately, 618 elderly patients with multimorbidity were enrolled in this study (309 in the intervention group and 309 in the control group) after propensity score matching. Considering the drop-out rates of 10%, the sample size still meets the calculation requirements. The flow chart of implementation effect research of the Rational Medication Management Mode is illustrated in Figure 2.

This study was approved by the Ethics Committee of School of Public Health, Fudan University (International Registration Number: IRB00002408 & FWA00002399; approval number: IRB#2021-11-0931).

### Patients

#### Inclusion criteria

Referring to the screening criteria of relevant modes (20), this study included elderly patients with high demand for medication management services and the ability to collaborate in the implementation process. The inclusion criteria are as follows: (*i*) aged 65 ~ 75 years old; (*ii*) suffering from 2 or more common chronic diseases currently (hypertension, coronary heart disease, stroke, diabetes, chronic obstructive pulmonary disease, osteoporosis, *etc.*,); (*iii*) taking 2 or more drugs (including over-the-counter drugs); *iv*) require long-term medication; *v*) the condition is stable without acute symptoms; (*vi*) having health records in the community healthcare center and continuously visiting and prescribing drugs for more than 3 months; (*vii*) voluntarily participate in intervention projects and obtain informed consent.

#### Exclusion criteria

Patients with the following conditions were excluded: *(i*) consciousness disorders or communication difficulties; (*ii*) currently using medication reminder software; *(iii*) unstable condition; (*iv*) those who have plans to go out in the last 3 months, and the time of going out is more than 1 month; (*v*) not suitable to participate in this study from a specialist point of view (such as incapacity).

#### Elimination criteria

Those participants who dropped out and lost follow-up were excluded. If the subjects did not arrive at the community healthcare center within 3 months and could not be contacted, they were regarded as lost to follow-up.

### Outcomes

The primary outcome of this study was medication number and polypharmacy (taking 5 or more medications) at 90 days.

The secondary outcomes included the situation for behavioral self-management and knowledge-belief-behavior of rational medication use. The behavioral self-management included the indicators of (*i*) medication reminders and its methods, (*ii*) intermittent medication and its reasons, and (*iii*) occurrence and handling of adverse drug reactions (ADRs).

The adapted scales were used for the evaluation of knowledge-belief-behavior for the rational medication (see Supplementary Table 2) (21, 22). According to the pre-survey, the rational medication knowledge scale and the medication behavior security scale optimized the expressions of the existing scales questions based on the opinions of the respondents. The rational medication belief scale and the medication behavior compliance scale were adapted from the existing scales, and the reliability and validity were tested based on the pre-survey. Cronbach's α coefficient was used to test the reliability of the scale, which demonstrated good consistency and reliability (Cronbach's α values were all > 0.7). Factorial analysis was used to test the validity of the scale (Kaiser-Meyer-Olkin value = 0.672; Bartlett's test of sphericity *P* value < 0.001), the cumulative variance interpretation rate was 67.30% when the eigenvalue is > 1, indicating good validity.

### Statistical analysis

IBM SPSS Statistics 23.0 was used for data analysis. The data normality test was performed on the measurement data; the mean ± standard deviation was used to describe the measurement data with normal distribution; the quartiles were used to describe the measurement data with non-normal distribution; the composition ratio (%) was used to describe the count data. The differences between groups were analyzed by *t*-test for normally distributed measures, non-parametric tests for non-normally distributed measures, and chi-square test and Fisher exact probability method for counting data. A *P* value < 0.05 was considered significant.

## Results

### Baseline characteristics for elderly patients with multimorbidity

A total of 618 elderly patients (309 in the intervention group and 309 in the control group) with multimorbidity were included in this study, those were all available at follow-up at 90 days. Baseline characteristics of demographic and health status of the study participants are presented in Table 1.

**Table 1:** Baseline characteristics of elderly patients with multimorbidity.

**Variable**	**Total (*n* = 618)**	**Intervention group (*n* = 309)**	**Control group (*n* = 309)**	***P* value**
Age (year)^*^	70.26 (4.23)	70.33 (4.28)	70.18 (4.19)	0.67
**Sex**				1.00
Male	306 (49.51)	153 (49.52)	153 (49.52)	
Female	312 (50.49)	156 (50.48)	156 (50.48)	
**Living areas**				0.47
Central-city	296 (47.90)	153 (49.52)	143 (46.28)	
Countryside	322 (52.10)	156 (50.48)	166 (53.72)	
**Marital status**				0.79
Married	557 (90.13)	280 (90.62)	277 (89.64)	
Unmarried	61 (9.87)	29 (9.38)	32 (10.36)	
**Education**				0.44
Primary school or lower	239 (38.67)	124 (40.13)	115 (37.22)	
Middle school	162 (26.21)	72 (23.30)	90 (29.12)	
High school	160 (25.89)	83 (26.86)	77 (24.92)	
University or higher	57 (9.22)	30 (9.71)	27 (8.74)	
**Living pattern**				0.64
Living alone	47 (7.61)	20 (6.47)	27 (8.74)	
Living with spouse	399 (64.56)	198 (64.08)	201 (65.05)	
Living with children	80 (12.95)	43 (13.92)	37 (11.97)	
Living with spouse and children	92 (14.89)	48 (15.53)	44 (14.24)	
**Household income per capita monthly (USD$)**				0.55
<150	2 (0.32)	0 (0.00)	2 (0.65)	
150 ~ 449	176 (28.48)	88 (28.48)	88 (28.48)	
450 ~ 749	220 (35.60)	109 (35.27)	111 (35.92)	
≥ 750	220 (35.60)	112 (36.25)	108 (34.95)	
**Basic medical insurance type**				0.01
For urban workers	240 (38.83)	136 (44.01)	104 (33.66)	
For residents	378 (61.17)	173 (55.99)	205 (66.34)	
Number of disease^*^	2.80 (0.93)	2.73 (0.85)	2.87 (1.00)	0.06
**Main disease**				
Hypertension	531 (85.92)	265 (85.76)	266 (86.08)	1.00
Diabetes	329 (53.24)	165 (53.40)	164 (53.07)	1.00
Coronary heart disease	178 (28.80)	92 (29.77)	86 (27.83)	0.66
Hyperlipidemia	138 (22.33)	66 (21.36)	72 (23.30)	0.63
Cerebral infarction	110 (17.80)	55 (17.80)	55 (17.80)	1.00
Number of medication^*^	4.25 (1.66)	4.17 (1.68)	4.33 (1.64)	0.23

* The data in this marked part are presented as mean (SD), and the others as frequencies (%).

There was no significant difference between the intervention group and the control group in age, gender, living area, marital status, education, living pattern, household income per capita monthly, the number of diseases, and the number of medications (*P* > 0.05). There was a statistical difference in the type of medical insurance between the two groups (*P* = 0.01). The homogeneity of baseline characteristics for the two groups is strong, especially the indicators of disease and medication, which were comparable.

### Primary outcome: Medication number and polypharmacy at 90 days

At 90 days, the number of medications was achieved by 3.88 (1.48) in the intervention group, while 4.32 (1.87) in the control group, which had a significant difference between the two groups (*P* = 0.001) (Table 2). And patients with polypharmacy were reduced by 59.55% in the intervention group at 90 days, having a significant difference compared with the control group (*P* < 0.001). The Rational Medication Management Mode had a positive effect on medication reconciliation (*P* = 0.024), while the conventional medication management was less effective (*P* = 0.91). Data on indicators are presented as the absolute change and percent change at 90 days from the baseline in each table.

**Table 2:** The number of medication and polypharmacy of elderly patients with multimorbidity.

**Primary outcome**	**Intervention group (*n* = 309)**	**Control group (*n* = 309)**	*P* value^*^
**Number of medication**			
Baseline	4.17 (1.68)	4.33 (1.64)	0.23
At 90 days	3.88 (1.48)	4.32 (1.87)	0.001
Absolute change	−0.29 (0.87)	−0.02 (1.17)	0.001
Percent change (%)	−0.05 (0.17)	0.01 (0.29)	0.001
*P* value^†^	0.024	0.91	-
**Polypharmacy (taking 5 or more medications) (%)**			
Baseline	269 (87.06)	105 (33.98)	<0.0001
At 90 days	85 (27.51)	112 (36.25)	0.025
*P* value^†^	<0.0001	0.61	-

* *P* value means the differences between two groups.

† *P* value means the differences between before baseline and after follow-up.

### Secondary outcomes: Behavioral self-management

At 90 days, patients with medication reminders were achieved in 294 (95.15%) and 182 (58.90%) in the intervention group and control group respectively, which had a significant difference between the two groups (*P* < 0.001) (Table 3). For the methods of medication reminders, the main way in the intervention group was to use a pillbox (75.41%), while in the control group was to put drugs in conspicuous positions (42.72%).

**Table 3:** Medication reminders and its methods for elderly patients with multimorbidity.

**Measures (%)**	**Intervention group (*n* = 309)**	**Control group (*n* = 309)**	*P* value^*^
**Medication reminders**			
Baseline	145 (46.93)	171 (55.34)	0.04
At 90 days	294 (95.15)	182 (58.90)	<0.0001
*P* value^†^	<0.0001	0.42	-
**Methods of medication reminders**			
**Follow daily activities**			
Baseline	43 (13.92)	58 (18.77)	0.13
At 90 days	8 (2.59)	25 (8.09)	0.004
*P* value^†^	<0.0001	<0.0001	-
**Put drugs in conspicuous positions**			
Baseline	70 (22.65)	89 (28.80)	0.10
At 90 days	169 (54.69)	132 (42.72)	0.004
*P* value^†^	<0.0001	<0.0001	-
**Mobile phone alarm clock**			
Baseline	5 (1.62)	12 (3.88)	0.14
At 90 days	17 (5.50)	16 (5.18)	1.00
*P* value^†^	0.02	0.56	-
**Family members help remind**			
Baseline	27 (8.74)	43 (13.92)	0.06
At 90 days	81 (26.21)	26 (8.41)	<0.0001
*P* value^†^	<0.0001	0.04	-
**Using pillbox**			
Baseline	20 (6.47)	8 (2.59)	0.03
At 90 days	233 (75.41)	35 (11.33)	<0.0001
*P* value^†^	<0.0001	<0.0001	-
**Medication notes**			
Baseline	9 (2.91)	5 (1.62)	0.42
At 90 days	92 (29.77)	9 (2.91)	<0.0001
*P* value^†^	<0.0001	0.42	-

* *P* value means the differences between two groups.

† *P* value means the differences between before baseline and after follow-up.

Patients with intermittent medication were reduced to 47 (15.21%) from 148 (47.90%) in the intervention group at 90 days, having a significant difference compared with the control group (*P* < 0.001) (Table 4). For the reason of reduction in intermittent medication, there were better improvements in poor memory, variety of drugs, withdrawal on their own, *etc*. in the experimental group (*P* < 0.05).

**Table 4:** Intermittent medication and its reasons for elderly patients with multimorbidity.

**Measures (%)**	**Intervention group (*n* = 309)**	**Control group (*n* = 309)**	*P* value^*^
**Intermittent medication**			
Baseline	148 (47.90)	150 (48.54)	0.94
At 90 days	47 (15.21)	131 (42.40)	<0.0001
*P* value^†^	<0.0001	0.15	-
**Reason for intermittent medication**			
**Poor memory**			
Baseline	82 (26.54)	73 (23.63)	0.46
At 90 days	30 (9.71)	65 (21.04)	<0.0001
*P* value^†^	<0.0001	0.50	-
**Variety of medications**			
Baseline	58 (18.77)	50 (16.18)	0.46
At 90 days	24 (7.77)	51 (16.51)	0.001
*P* value^†^	<0.0001	1.00	-
**Going out without drugs**			
Baseline	38 (12.30)	31 (10.03)	0.44
At 90 days	12 (3.88)	41 (13.27)	<0.0001
*P* value^†^	<0.0001	0.26	-
**Not renewing the prescription in time**			
Baseline	33 (10.68)	37 (11.97)	0.70
At 90 days	10 (3.24)	45 (14.56)	<0.0001
*P* value^†^	<0.0001	0.41	-
**Withdrawal on their own**			
Baseline	25 (8.09)	25 (8.09)	1.00
At 90 days	10 (3.24)	18 (5.83)	0.17
*P* value^†^	0.01	0.34	-

* *P* value means the differences between two groups.

† *P* value means the differences between before baseline and after follow-up.

At 90 days, patients who occurred on ADRs were reduced to 51 (16.51%) from 88 (28.48%) in the intervention group, having a significant difference compared with the control group (*P* = 0.01) (Table 5). For the handling of ADRs, the way to ask family physicians increased remarkably in the intervention group (*P* < 0.0001).

**Table 5:** Occurrence and handing of adverse drug reactions (ADR) of elderly patients with multimorbidity.

**Measures (%)**	**Intervention group (*n* = 309)**	**Control group (*n* = 309)**	*P* value^*^
**ADRs occurred**			
Baseline	88 (28.48)	93 (30.10)	0.72
At 90 days	51 (16.51)	78 (25.24)	0.01
*P* value^†^	<0.0001	0.21	-
**Handling of ADRs**			
**Asking the family physician as soon as possible**			
Baseline	144 (46.60)	161 (52.10)	0.20
At 90 days	194 (62.78)	145 (46.93)	<0.0001
*P* value^†^	<0.0001	0.23	-
**Asking the family physician on next visit**			
Baseline	178 (57.61)	188 (60.84)	0.46
At 90 days	238 (77.02)	220 (71.20)	0.12
*P* value^†^	<0.0001	0.008	-
**Asking family members**			
Baseline	75 (24.27)	78 (25.24)	0.85
At 90 days	120 (38.84)	125 (40.45)	0.74
*P* value^†^	<0.0001	<0.0001	-
**Reading the instructions and handling them on their own**			
Baseline	50 (16.18)	73 (23.63)	0.03
At 90 days	37 (11.97)	91 (29.45)	<0.0001
*P* value^†^	0.16	0.12	-
**Checking the experience online**			
Baseline	11 (3.56)	12 (3.88)	1.00
At 90 days	4 (1.29)	13 (4.21)	0.04
*P* value^†^	0.11	1.00	-
**No handling**			
Baseline	8 (2.59)	11 (3.56)	0.64
At 90 days	0 (0.00)	6 (1.94)	0.03
*P* value^†^	0.007	0.33	-

* *P* value means the differences between two groups.

† *P* value means the differences between before baseline and after follow-up.

### Secondary outcomes: Knowledge-belief-behavior evaluation

The knowledge, belief, behavior security and behavior compliance of rational medication use of elderly patients were all greatly improved in the intervention group at 90 days (*P* < 0.0001), which was statistically different compared with the control group (*P* < 0.0001), explaining the Rational Medication Management Mode had a positive effect on health education for elderly patients. Data on these measures are presented as the absolute change and percent change at 90 days from the baseline in Tables 6–9.

**Table 6:** The rational medication knowledge evaluation of elderly patients with multimorbidity.

**Measures**	**Intervention group (*n* = 309)**	**Control group (*n* = 309)**	*P* value^*^
**Total points**			
Baseline^#^	19.61 (7.73)	20.21 (8.23)	0.35
At 90 days^#^	28.33 (6.42)	22.18 (7.70)	<0.0001
Absolute change^#^	8.72 (8.67)	1.97 (8.67)	<0.0001
Percent change (%)	0.68 (0.85)	0.39 (1.34)	<0.0001
*P* value^†^	<0.0001	0.002	-
**Ranking points (%)**			
**High level (29–36 points)**			
Baseline	36 (11.65)	57 (18.45)	0.02
At 90 days	175 (56.63)	73 (23.63)	<0.0001
*P* value^†^	<0.0001	0.14	-
**Medium level (20–28 points)**			
Baseline	118 (38.19)	112 (36.25)	0.68
At 90 days	109 (35.28)	131 (42.40)	0.08
*P* value^†^	0.50	0.14	-
**Low level (0–19 points)**			
Baseline	155 (50.16)	140 (45.31)	0.26
At 90 days	25 (8.09)	105 (33.98)	<0.0001
*P* value^†^	<0.0001	0.005	-

* *P* value means the differences between two groups.

† *P* value means the differences between before baseline and after follow-up.

# The data in this marked part are presented as mean (SD), and the others as frequencies (%).

**Table 7:** The rational medication belief evaluation of elderly patients with multimorbidity.

**Measures**	**Intervention group (*n* = 309)**	**Control group (*n* = 309)**	*P* value^*^
**Total points**			
Baseline^#^	3.98 (1.80)	4.00 (1.67)	0.87
At 90 days^#^	4.77 (1.89)	3.82 (1.63)	<0.0001
Absolute change^#^	0.79 (2.15)	−0.18 (2.03)	<0.0001
Percent change (%)	0.39 (0.97)	0.13 (0.77)	<0.0001
*P* value^†^	<0.0001	0.18	-
**Ranking points (%)**			
**High level (6–7 points)**			
Baseline	55 (17.80)	62 (20.07)	0.54
At 90 days	131 (42.40)	57 (18.45)	<0.0001
*P* value^†^	<0.0001	0.68	-
**Medium level (4–5 points)**			
Baseline	135 (43.69)	126 (40.78)	0.52
At 90 days	107 (34.63)	112 (36.25)	0.74
*P* value^†^	0.03	0.28	-
**Low level (0–3 points)**			
Baseline	119 (38.51)	121 (39.16)	0.93
At 90 days	71 (22.98)	140 (45.31)	<0.0001
*P* value^†^	<0.0001	0.14	-

* *P* value means the differences between two groups.

† *P* value means the differences between before baseline and after follow-up.

# The data in this marked part are presented as mean (SD), and the others as frequencies (%).

**Table 8:** The medication behavior security evaluation of elderly patients with multimorbidity.

**Measures**	**Intervention group (*n* = 309)**	**Control group (*n* = 309)**	*P* value^*^
**Total points**			
Baseline^#^	7.60 (2.45)	7.17 (2.14)	0.02
At 90 days^#^	8.80 (1.95)	7.53 (2.17)	<0.0001
Absolute change^#^	1.20 (2.47)	0.37 (2.05)	<0.0001
Percent change (%)	0.39 (1.12)	0.12 (0.47)	<0.0001
*P* value^†^	<0.0001	0.03	-
**Ranking points (%)**			
**Relatively safe (10–11 points)**			
Baseline	82 (26.54)	41 (13.27)	<0.0001
At 90 days	165 (53.40)	63 (20.39)	<0.0001
*P* value^†^	<0.0001	0.02	-
**Inappropriate use (7–9 points)**			
Baseline	150 (48.54)	154 (49.84)	0.81
At 90 days	112 (36.25)	166 (53.72)	<0.0001
*P* value^†^	0.003	0.38	-
**Dangers (0–6 points)**			
Baseline	77 (24.92)	114 (36.89)	0.002
At 90 days	32 (10.36)	80 (25.89)	<0.0001
*P* value^†^	<0.0001	0.004	-

* *P* value means the differences between two groups.

† *P* value means the differences between before baseline and after follow-up.

# The data in this marked part are presented as mean (SD), and the others as frequencies (%).

**Table 9:** The medication behavior compliance evaluation of elderly patients with multimorbidity.

**Measures**	**Intervention group (*n* = 309)**	**Control group (*n* = 309)**	*P* value^*^
**Total points**			
Baseline^#^	8.60 (2.84)	8.35 (2.68)	0.27
At 90 days^#^	10.82 (1.90)	8.83 (2.42)	<0.0001
Absolute change^#^	2.22 (2.86)	0.48 (2.54)	<0.0001
Percent change (%)	0.39 (0.55)	0.16 (0.57)	<0.0001
*P* value^†^	<0.0001	0.02	-
**Ranking points (%)**			
**High level (10–12 points)**			
Baseline	121 (39.16)	106 (34.30)	0.24
At 90 days	240 (77.67)	118 (38.19)	<0.0001
*P* value^†^	<0.0001	0.36	-
**Medium level (8–9 points)**			
Baseline	103 (33.33)	107 (34.63)	0.80
At 90 days	50 (16.18)	131 (42.40)	<0.0001
*P* value^†^	<0.0001	0.06	-
**Low level (0–7 points)**			
Baseline	85 (27.51)	96 (31.07)	0.38
At 90 days	19 (6.15)	60 (19.42)	<0.0001
*P* value^†^	<0.0001	0.001	-

* *P* value means the differences between two groups.

† *P* value means the differences between before baseline and after follow-up.

# The data in this marked part are presented as mean (SD), and the others as frequencies (%).

## Discussion

With the acceleration of population aging and the changing in disease spectrum, the long-term medication demand of patients with chronic diseases is increasing (23). More practical research is required to evaluate the implementation effect of technology/tool-based mode and ensure they meet their potential and value in improving chronic disease management and rational medication use for patients (24).

The medication administration mode, which was widely used in countries with mature medication administration experience, comprised of drug administration tools, such as medication record form in the United States, the drug administration manual in Japan, *etc.*, The effectiveness of medication management tools in improving patient compliance and health outcomes has been confirmed in relevant studies. A survey conducted by the Japanese Ministry of Health, Labor and Welfare showed that 50 ~ 90% of citizens of different ages were using the drug administration manual (25). A study has shown that the protocol-based pharmacotherapy management (PBPM) mode can effectively reduce the burden of doctors and nurses, and improve the safety of drug therapy for patients (26). Nevertheless, in the practice of medication management in China, there is a lack of tools to integrate patients' medication information. The existing medical records, health records, information systems, *etc*. are mostly used by medical staff, which is difficult to achieve joint records between doctors and patients, especially lacking ways for patients to report home medication situations. Based on the framework of medication record tools in the United States and Japan, and the medication management needs of the elderly, this study designed a tool of medication management manual for the elderly, which became the core technical support for the Rational Medication Management Mode.

Ensuring medication safety in polypharmacy is one of the key challenges for medication safety today. All stakeholders, including physicians, nurses, pharmacists, and even patients and their families, could have a vital role to play in driving change for the management of multiple drugs. Patients should be viewed as shared decision-makers on the use of medication, and health care professionals need to encourage and support patients and their families to disclose all the medications they are taking, including over-the-counter, traditional and complementary medicines, especially if they are suffering from multiple conditions and are being treated with polypharmacy (27–29). Family physicians and pharmacists should conduct medication reviews and medication reconciliation whenever possible in collaboration with the patient and their families, which has a positive impact on optimizing medicine use and improving health outcomes (30–32). The effect evaluation of this study found that after the implementation of Rational Medication Management Mode, the number of medications and the proportion of polypharmacy in the intervention group decreased significantly at 90 days, having a significant difference compared with the control group (*P* < 0.001).

Elderly chronic diseases and medication management need to pay attention to the establishment of an efficient interactive relationship with patients, strengthening the persistence of drug records and the self-management ability of patients (33–35). For example, family physicians and pharmacists help patients with complex medication regimens to clarify the usage and dosage of all kinds of drugs, reducing the occurrence of intermittent medication due to a variety of medications; patients are required to check the corresponding position after taking medication every day, helping to establish medication habits; dispensing into the pillbox can help patients take their medicines with them when they go out. The effect evaluation that, the proportion of elderly patients with medication reminders increased significantly, and the numbers of intermittent medication and ADR decreased significantly (*P* < 0.05).

If the elderly have weak awareness of rational drug use, it is hard to fully realize the value of medication management to improve their health status. Health education for patients plays a key role in the prevention and early detection of medication use and related health problems by raising their awareness of irrational drug use (4, 36–38). The knowledge-belief-behavior evaluation of rational medication use for patients can reflect the practical effect of the mode in health education (39). This study found that this mode can better improve patients' knowledge and belief in rational medication, and improve the security and compliance of medication behavior (*P* < 0.0001). It is worth noting that, for the evaluation of knowledge and behavior compliance, the proportion of low levels in the control group is significantly reduced compared with the baseline (*P* < 0.05), while there is no statistical difference in high levels. The evaluation of medication behavior security also showed a similar situation, the reduction degree of dangers level was greater than the improvement degree of the relatively safe level. This indicates that with the improvement of living standards and various health education, elderly patients began to enhance their awareness level of medication use, paying attention to knowledge learning and behavior improvement. However, in this process, it is difficult for elderly patients to find a scientific path of improvement by relying on their own experience and learning. Rational Medication Management Mode based on family physician services can help the elderly to learn drug use knowledge and adopt healthy behavior scientifically, and improve the long-term management efficiency of family physician teams, to maximize health effect gain and reduce the disease burden.

This study still has some limitations. First, some information on secondary outcomes was obtained through the self-reported questionnaire, which may constitute a reporting bias. However, this is likely to be an undifferentiated bias. Second, the experimental time of this study is short. And the mode needs certain application conditions, such as economically developed areas, mature family physician services, pharmacist equipment. However, after the effectiveness of this mode has been confirmed, we have begun to pilot promotion, and started to optimize the applicability of the mode, attempting to promote and apply it to other regions and key populations.

## Conclusion

To sum up, this study constructed a Rational Medication Management Mode and carried out an empirical research to evaluate the implementation effect. The mode-based family physician service, which provides the support of manuals and pillboxes, can decrease the drug numbers of elderly patients with multimorbidity, reduce the incidence of polypharmacy, enhance behavioral self-management, increase the knowledge and belief of rational medication use, and improve the security and compliance of medication use behavior. In order to provide a practical basis for rational medication management of elderly patients with multimorbidity under the background of long-term prescriptions in China.

## Data availability statement

The datasets presented in this article are not readily available because the datasets generated during and/or analyses during the current study are available from the authors on reasonable request. Requests to access the datasets should be directed to QT, tangqi@fudan.edu.cn.

## Ethics statement

The studies involving human participants were reviewed and approved by the Ethics Committee of School of Public Health, Fudan University (International Registration Number: IRB00002408 & FWA00002399; approval number: IRB#2021-11-0931). The patients/participants provided their written informed consent to participate in this study.

## Author contributions

JuL and GC conceived and designed the research and critically reviewed the manuscript. QT analyzed the data, wrote the initial manuscript and generated the tables and figures. LW revised the manuscript critically for important intellectual content. JiL, WW, and ZL helped in data collecting. SZ proofread the manuscript. CL supervised the research. All authors approved the final version of the paper and take responsibility for its content. All authors contributed to the article and approved the submitted version.

## Funding

This study was supported by the Major Project of National Social Science Foundation of China (Grant No. 17ZDA078), National Natural Science Foundation of China (Grant No. 72004030) and Policy Research Project of Shanghai Municipal Health Commission (Grant No. 2022HP53).

## Conflict of interest

The authors declare that the research was conducted in the absence of any commercial or financial relationships that could be construed as a potential conflict of interest.

## Publisher's note

All claims expressed in this article are solely those of the authors and do not necessarily represent those of their affiliated organizations, or those of the publisher, the editors and the reviewers. Any product that may be evaluated in this article, or claim that may be made by its manufacturer, is not guaranteed or endorsed by the publisher.
