# Supplementary material for: Rational medication management mode and its implementation effect for the elderly with multimorbidity: A prospective cohort study in China
Source: Front Public Health. 2022 Sep 6;10:992959. doi: 10.3389/fpubh.2022.992959 (PMC9486462; doi:10.3389/fpubh.2022.992959)
Supplement: Supplementary file 1 [file Table_1.DOCX]

**Supplementary Table 1. Framework and contents of medication management manual for elderly*.**

| **First level directory** | **Second level directory** | **Contents** |
| --- | --- | --- |
| A Basic Information | A1 personal | A1_1 name |
|  |  | A1_2 age |
|  |  | A1_3 sex |
|  |  | A1_4 height |
|  |  | A1_5 weight |
|  |  | A1_6 contact number |
|  | A2 emergency contact | A2_1 name |
|  |  | A2_2 relationship |
|  |  | A2_3 contact number |
|  | A3 family physician | A3_1 name |
|  |  | A3_2 work units |
|  |  | A3_3 contact number |
|  | A4 diagnosis and treatment | A4_1 current disease type |
|  |  | A4_2 years of illness |
|  |  | A4_3 ADRs history |
|  |  | A4_4 food allergic history |
|  |  | A4_5 other allergies |
|  |  | A4_6 operation History |
| B Medications List | B1 drug generic name | — |
|  | B2 starting date | — |
|  | B3 expiration date | — |
|  | B4 major functions | — |
|  | B5 usage and dosage | B5_1 single dose |
|  |  | B5_2 daily dose |
|  |  | B5_3 medication time |
|  | B6 medication guidance | — |
| C Health Care Products List | C1 name | — |
|  | C2 starting date | — |
|  | C3 expiration date | — |
|  | C4 major functions | — |
|  | C5 usage and dosage | — |
|  | C6 source | — |
| D Medications Record Weekly | D1 drug generic name | — |
|  | D2 week | D2_1 Monday/Tuesday/Wednesday... |
|  | D3 ADRs | — |
|  | D4 special situation | — |
| E Medications Record Evaluation Monthly | E1 self-reported medication compliance | — |
|  | E2 needs to consult physicians | — |
|  | E3 medication management evaluation | — |
|  | E4 medication guidance | — |
|  | E5 recommended follow-up time | — |
| F Health-related Index Record Weekly | F1 recording time | — |
|  | F2 blood pressure | — |
|  | F3 heart rate | — |
|  | F4 weight | — |
|  | F5 special situation | — |
| G More About | G1 introduction to the manual | — |
|  | G2 user guide | — |
|  | G3 propaganda and education of medication knowledge | — |

*The original version used in the study is in Chinese, presented as the translated version in English in the supplementary table.
