# Supplementary material for: Rational medication management mode and its implementation effect for the elderly with multimorbidity: A prospective cohort study in China
Source: Front Public Health. 2022 Sep 6;10:992959. doi: 10.3389/fpubh.2022.992959 (PMC9486462; doi:10.3389/fpubh.2022.992959)
Supplement: Supplementary file 2 [file Table_2.DOCX]

**Supplementary Table 2. The scales of knowledge-belief-behavior for rational medication use*.**

| **(A) Measures of rational medication knowledge** | **know nothing**  **(0 points)** | **know a little**  **(1 point)** | **know partly**  **(2 points)** | **know completely**  **(3 points)** |
| --- | --- | --- | --- | --- |
| 1. Do you know what medications you are taking every day? |  |  |  |  |
| 1. Do you know the expiration date of the medications you are taking? |  |  |  |  |
| 1. Do you know the function of the medications you are taking? |  |  |  |  |
| 1. Do you know the usage and dosage of the medications you are taking? |  |  |  |  |
| 1. Do you know when you should take medications every day? |  |  |  |  |
| 1. Do you know the side effects of the medications you are taking? |  |  |  |  |
| 1. Do you know the contraindications of the medications you are taking? |  |  |  |  |
| 1. Do you know the instructions for taking medications given by the doctor? |  |  |  |  |
| 1. Do you know how to store medications correctly? |  |  |  |  |
| 1. Do you know what to do with the medications that can not taking? |  |  |  |  |
| 1. Do you know how to identify fake and inferior medications? |  |  |  |  |
| 1. Do you know the physiological characteristics and medication particularity of the elderly? |  |  |  |  |
| 1. **Measures of rational medication belief** | | **yes**  **(0 points)** | **no**  **(1 point)** | **uncertainty**  **(0 points)** |
| 1. Do you think it is not important for patients with chronic diseases to measure and record blood pressure and blood glucose every day for disease control? | |  |  |  |
| 1. Do you think the therapeutic effect of imported medications is better than that of domestic drugs? | |  |  |  |
| 1. Do you think that if you take several medications with good curative effects together, the therapeutic effect will be better? | |  |  |  |
| 1. Do you think that the side effects of traditional Chinese medicines are smaller than those of western medications, and you can always eat them in order to regulate your body? | |  |  |  |
| 1. Do you think health products can be taken as medications? | |  |  |  |
| 1. Do you think you only need to follow the doctor's instructions in the process of taking medications, and you don't need to participate in medication management? | |  |  |  |
| 1. Do you think chronic diseases can be effectively controlled if you persist in taking medications and maintain good living habits? | |  |  |  |
| 1. **Measures of medication behavior security** | | | **yes**  **(0 points)** | **no**  **(1 point)** |
| 1. When you feel your condition is stable or under control, will you stop taking the medications temporarily or reduce the dosage? | | |  |  |
| 1. Will you judge your state based on your feelings and then decide whether you need to take medications, rather than judging based on the blood pressure and blood glucose measurement results? | | |  |  |
| 1. Do you usually take medications irregularly and take them once when you remember? | | |  |  |
| 1. Will you hoard the medications that you haven't finished eating once, so that you can take them out and eat them next time you get sick? | | |  |  |
| 1. Have you ever taken expired medications? | | |  |  |
| 1. In order to see different diseases, will you go to different specialized hospitals to see a specialist and prescribe medications? | | |  |  |
| 1. Will you stop taking a medication or take another kind of medication without consulting a doctor because you haven't gotten better for a few days? | | |  |  |
| 1. Will you take fewer medications because you worry about side effects? | | |  |  |
| 1. If you forget to take medications once, will you take more medications next time? | | |  |  |
| 1. If someone has taken or heard of a medication with good effect recommended to you, will you buy it? | | |  |  |
| 1. If you see a medication that works well when watching TV or advertisements, will you buy it? | | |  |  |
| 1. **Measures of medication behavior compliance** | **completely unable**  **(0 points)** | **occasionally could**  **(1 point)** | **basically could**  **(2 points)** | **completely could**  **(3 points)** |
| 1. Can you take medications according to the number of times required by your doctor or pharmacist every day? |  |  |  |  |
| 1. Can you take medications according to the dosage required by your doctor or pharmacist every day? |  |  |  |  |
| 1. Can you take medications regularly according to the time required by the doctor or pharmacist? |  |  |  |  |
| 1. Can you take medications continuously for a long period of time according to the requirements of your doctor or pharmacist? |  |  |  |  |

*The original version used in the study is in Chinese, presented as the translated version in English in the supplementary table.

1. Total points of 29 ~ 36 means high level; of 20 ~ 28 means medium level; of 0 ~ 19 means low level.
2. Total points of 6 ~7 means high level; of 4 ~ 5 means medium level; of 0 ~ 3 means low level.
3. Total points of 10 ~ 11 means relatively safe; of 7 ~ 9 means inappropriate use; of 0 ~ 6 means dangers.
4. Total points of 10 ~12 means high level; of 8 ~ 9 means medium level; of 0 ~ 7 means low level.
